# Supplementary material for: From eggs to guts: Symbiotic association of Sodalis nezarae sp. nov. with the Southern green shield bug Nezara viridula
Source: FEMS Microbiol Ecol. 2025 Feb 12;101(3):fiaf017. doi: 10.1093/femsec/fiaf017 (PMC11879575; doi:10.1093/femsec/fiaf017)
Supplement: fiaf017_Supplemental_Files [file fiaf017_supplemental_files.zip › Sodalis_Supplementary_Figures.docx]

**Supplementary Figures**


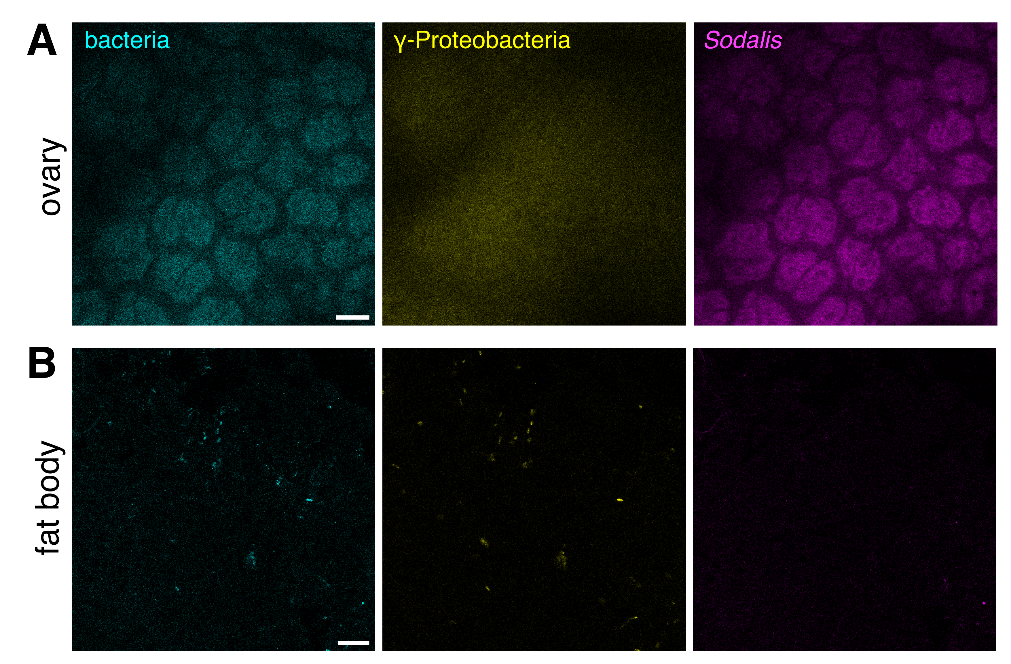


**Supplementary Figure 1. Colonization of Nezara viridula ovaries and fat body surface by Gammaproteobacteria and Sodalis.** Confocal micrographs show **A)** the adult N. viridula ovary, scale bar = 10 µm and **B)** the surface of the fat body, scale bar = 10 µm. Panel bacteria shows FISH-probe Eub-mix in cyan (Fluos). Panel γ-Proteobacteria shows FISH-probe GAM42A in yellow (Cy5). Panel Sodalis shows FISH-probe Sod1238R in magenta (Cy3). The images shown here are representative of FISH micrographs collected from three female insects.


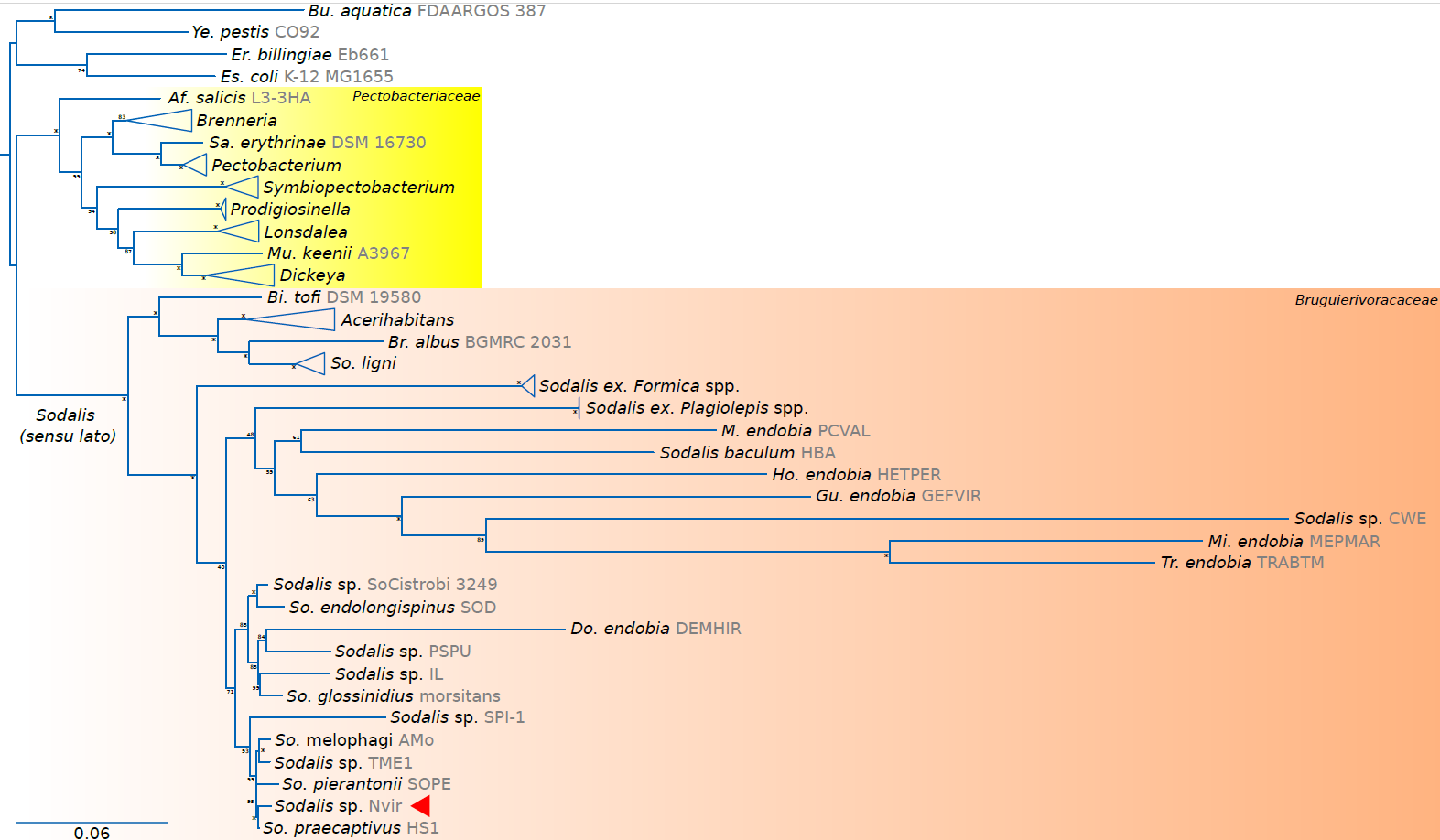


**Supplementary Figure 2. Phylogenetic relationships of Pectobacteriaceae and Bruguierivoracaceae genera.** Maximum-Likelihood phylogenetic tree as calculated using IQ-TREE v2.2.2.7 (LG4X+I+G; 1000 UltraFast bootstrap replicates). Names on leaves specify the bacterial genera, species, and strains (in grey). Coloured boxes delimit the Pectobacteriaceae and the proposed "Bruguierivoracaceae" (or Sodalis sensu lato). A red left-pointing arrow highlights the novel Sodalis sp. Nvir. Values at nodes indicate the UltraFast bootstrap support values in percentages. An asterisk (*) denotes a support of 100%.


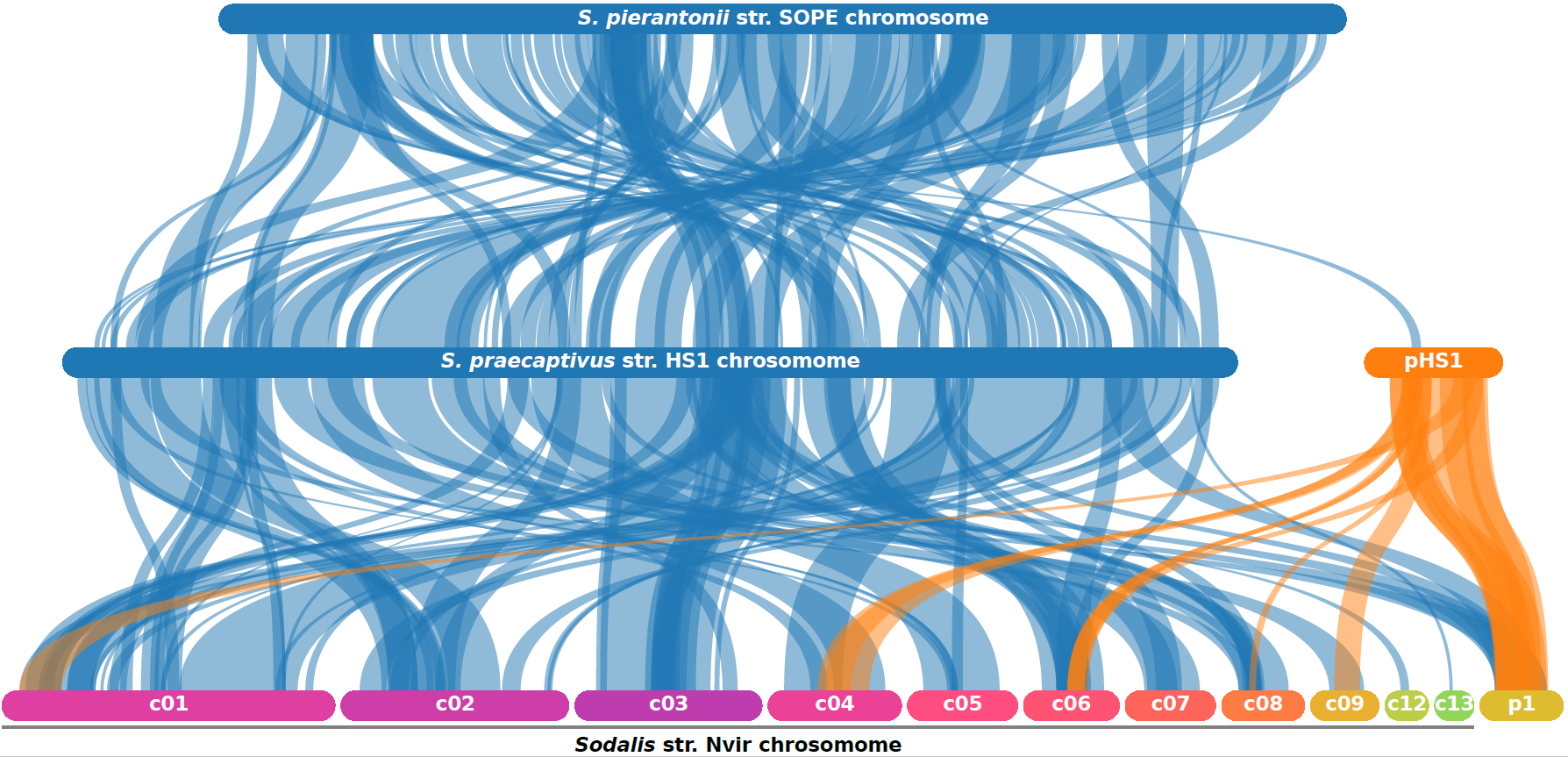


**Supplementary Figure 3. Syntenic blocks among selected Sodalis strains.** Coloured horizontal bars represent chromosomes/contigs, while vertical curved lines connect syntenic blocks. The latter are colour-coded in relation to the former. A "c" and "p" prefix in horizontal bar labels marks the molecule as a contig or a plasmid molecule, respectively. For simplicity, only molecules that share syntenic blocks are displayed. Horizontal bars are at scale in relation to the size of the molecule (in bps).
